# Implementing interventions to reduce work-related stress among health-care workers: an investment appraisal from the employer’s perspective

**DOI:** 10.1007/s00420-019-01471-y

**Published:** 2019-08-26

**Authors:** Ben F. M. Wijnen, Joran Lokkerbol, Cecile Boot, Bo M. Havermans, Allard J. van der Beek, Filip Smit

**Affiliations:** 1 Centre for Economic Evaluation, Trimbos-Institute, Netherlands Institute of Mental Health and Addiction, Utrecht, The Netherlands; 2 Department of Clinical Epidemiology and Medical Technology Assessment, Maastricht University Medical Centre, P.O. Box 616, 6200 Maastricht, The Netherlands; 3 Department of Public Mental Health, Trimbos-Institute, Netherlands Institute of Mental Health and Addiction, Utrecht, The Netherlands; 4 Department of Health Care Policy, Harvard Medical School, Boston, MA USA; 5 Department of Public and Occupational Health, Amsterdam Public Health Research Institute, VU University Amsterdam, Amsterdam UMC, Amsterdam, The Netherlands; 6 TNO-VU University Medical Centre, Body@Work, Research Centre Physical Activity, Work and Health, Amsterdam, The Netherlands; 7 Department of Epidemiology and Biostatistics, Amsterdam Public Health Research Institute, VU University Medical Centre, Amsterdam, The Netherlands

**Keywords:** Work stress, Prevention, Intervention, Employee perspective, Investment appraisal

## Abstract

**Purpose:**

The Stress-Prevention@Work implementation strategy has been demonstrated to be successful in reducing stress in employees. Now, we assess the economic return-on-investment to see if it would make for a favourable business case for employers.

**Methods:**

Data were collected from 303 health-care workers assigned to either a waitlisted control condition (142 employees in 15 teams) or to Stress-Prevention@Work (161 employees in 15 teams). Main outcome was productivity losses measured using the Trimbos and iMTA Cost questionnaire in Psychiatry. Measurements were taken at baseline, 6, and 12 months post-baseline.

**Results:**

The per-employee costs of the strategy were €50. Net monetary benefits were the benefits (i.e., improved productivity) minus the costs (i.e., intervention costs) and were the main outcome of this investment appraisal. Per-employee net benefits amounted to €2981 on average, which was an almost 60-fold payout of the initial investment of €50. There was a 96.7% likelihood for the modest investment of €50 to be offset by cost savings within 1 year. Moreover, a net benefit of at least €1000 still has a likelihood of 88.2%.

**Conclusions:**

In general, there was a high likelihood that Stress-Prevention@Work offers an appealing business case from the perspective of employers, but the employer should factor in the additional per-employee costs of the stress-reducing interventions. Still, if these additional costs were as high as €2981, then costs and benefits would break even.

This study was registered in the Netherlands National Trial Register, trial code: NTR5527.

## Background

Work-related stress is common in the health workforce (Ruotsalainen et al. 2008, 2015; Dharmawardene et al. 2016) and may compromise both health of the staff working at health services (Ganster and Rosen 2013; Steptoe and Kivimäki 2013) and the quality of the work for the patients they serve. After all, higher levels of stress are likely to translate into absenteeism from work (Henderson et al. 2005; Ruotsalainen et al. 2015) and reduced productivity while at work (Ganster and Rosen 2013; Steptoe and Kivimäki 2013). The corresponding productivity losses have economic implications for the employer of a health service (Hassard et al. 2014) and begs the question if implementing stress-reducing interventions at the workplace would represent a worthwhile investment as seen from the employer’s perspective (World Health Organization 2017).

Over the next 10–20 years, stress in the workforce for health is likely to increase, because health services will be increasingly stretched by an intensified demand for health care by Europe’s greying populations, yet the workforce in the health-care sector is ageing at the same rate (Dussault et al. 2010). Some labour market analysts predict that health services will find it increasingly difficult to attract new staff, which will then translate not only in an ageing, but also in a concomitant ageing (i.e., proportionally lower number of young employees) of the workforce for health (Lopes et al. 2015). The disparity between the increased demand for health care and dwindling numbers of ageing health-care workers will be one of Europe’s critical challenges (Roberfroid et al. 2009). Moreover, compared to other occupations, levels of dissatisfaction, distress and burnout at work are already relatively high in health care (McNeely 2005; Ruotsalainen et al. 2008, 2015).

Therefore, it becomes increasingly important to sustain the vitality of employees in the health-care sector. One strategy is to offer stress-reducing interventions at the workplace, but here we encounter an implementation problem: despite the abundance of evidence-based stress-reducing interventions (Noben et al. 2014, 2015) their implementation and uptake at the workplace is limited (Westgaard and Winkel 2011). A recent Cochrane review identified 58 studies which aimed to evaluate various interventions to prevent stress at work in health-care workers and concluded that, overall, the quality of the studies was poor and that more trials are needed (Ruotsalainen et al. 2015). Looking at organisational interventions (e.g., changes in working conditions, organising support, changing care, increasing communication skills and changing work schedule), 20 studies were included. However, most of these interventions were not more effective than no intervention or an alternative intervention. In addition, evidence regarding (health) economic benefits is lacking. Encouraging uptake requires a dedicated implementation strategy. To this end a portal, Stress-Prevention@Work, was developed to encourage teams of health-care workers to systematically review their own needs for stress management, select an appropriate intervention, and subsequently use and evaluate the intervention of their choice (Havermans et al. 2018b). Stress-Prevention@Work has been demonstrated to be successful in reducing stress in employees (Havermans et al. 2018a). Now, we used the empirical data to test the idea that implementing stress-reducing interventions for health-care workers offers good value for money as seen from the employer’s perspective. In other words, Stress-Prevention@Work might be associated with an appealing return-on-investment via the payout of increased productivity stemming from reduced absenteeism and presenteeism.

## Methods

### Participants and procedures

This study was conducted in a Dutch health service of 4500 employees. A total of 30 teams of health-care workers (mainly nurses) were recruited for participation and split into 15 teams (with 252 nurses) for the experimental condition where Stress-Prevention@Work was offered and matched with another 15 teams (with 221 nurses) as waitlisted controls. An independent researcher matched teams on working conditions and size and allocated the teams to the intervention or control group. This researcher did not have information about the perceived stress levels in the teams. Since the intervention focused on the organisation and not on individual workers, individual randomisation was not feasible. The main outcome of the study was productivity losses, assessed using the Trimbos and iMTA Cost questionnaire in Psychiatry (TiC-P), which is a self-completed questionnaire identifying days absent from work (absenteeism) and days working while sick (presenteeism). As we were specifically interested in productivity losses, only these items of the TiC-P were used (i.e. number of working hours per week, number of working days per week, number of days absent past months, number of days working while not fully fit). As the paper compromised an investment appraisal, we looked at both costs and (financial) benefits, which are described below. Measurements were conducted in May 2016 (baseline, *t*_0_) and then at 6 and 12 months post-baseline (*t*_1_ and *t*_2_). The control group was given access to the intervention after 12 months. The study design is therefore best described as a matched cohort study in two parallel groups with clustering at the team level. More details are provided in Hoek et al. (2018).

### Implementation strategy

Stress-Prevention@Work targeted all employees within the intervention groups and contained a search engine for interventions for stress prevention. The portal, and concomitant training in its usage, takes a stepwise approach directed at (1) raising awareness that proper management of work-related stress is important; (2) screening for determinants of work stress; (3) setting intervention goals and selecting appropriate preventive interventions; (4) implementing the selected stress management intervention in the workplace [e.g., construct a personal action plan to implement the intervention(s)]; and (5) evaluating the strategy’s impact on work-related stress. Types of interventions ranged from guidelines for communicating about work stress to more extensive, tailor-made interventions, possibly involving intermediaries/consultants. The interventions were either at organisational or employee level. For example, organisational interventions included a guideline to start a dialogue between employees and their manager(s) about the presence of work-related stress within the organisation or team. Individual interventions could consist of online self-help modules to reduce work-related psychosocial risk factors (Hoek et al. 2018).

### Computation of costs

This investment appraisal looked at the costs associated with the Stress-Prevention@Work implementation strategy and compared these costs with the economic benefits derived from greater work productivity through lesser absenteeism and presenteeism. On the costs side, the following items are included:

1. The per-team costs of using Stress-Prevention@Work is €100. This per-team tariff helps to pay for hosting, maintaining and periodically upgrading the Stress-Prevention@Work portal. In an average team of 16 health-care workers this amounts to €100/16 = €6.25 per employee.
2. In each team, one employee receives training in the Stress-Prevention@Work strategy at €250. In an average team of 16 nurses, this translates in an additional per-nurse cost of €250/16 = €15.63.
3. Each employee spends 30 min (at most) during office hours on working through all the steps of the Stress-Prevention@Work implementation strategy. This is equivalent to €17.40 per employee, when valuing 1 h of work of an employee at €34.75 (for the year 2014) in line with the Dutch guideline for costing in health-economic evaluation (Zorginstituut Nederland 2015).
4. However, there is one employee in each team who operates the portal and this takes 3 h at €34.75 per hour, which adds (3 × €34.75)/16 = €6.50 per employee.
5. Finally, the Human Resource Management department at the health service invests 150 h of work annually to promote the Stress-Prevention@Work strategy across all 4500 employees. This represents costs of (150 h × €34.75)/4500 = €1.60 per employee.

The sum total of costs of Stress-Prevention@Work is therefore 6.25 + 15.63 + 17.40 + 6.50 + 1.60 = €47.38 per employee per annum. This is rounded to €50 per employee. It is assumed that these costs are incurred by the employer. Moreover, it is assumed that more extensive interventions take place outside office hours.

### Computation of benefits

The economic benefits from Stress-Prevention@Work are generated by greater work productivity via lesser absenteeism and lesser presenteeism. As before, we value 1 h of work at €34.75. Thus, 1 day of sick leave costs 8 h × €34.75 = €278. The number of days of sick leave was estimated using the TiC-P at each of the three measurements points (*t*_0_, *t*_1_ and *t*_2_) and multiplied by €278.

In a similar vein, the costs of presenteeism were estimated as the cost of workdays lost due to presenteeism. The TiC-P helps to assess the number of lost workdays owing to presenteeism by first asking how many days the employee went to work not feeling well and then by asking to rate the efficiency at work while not feeling well. Thus, 2 days worked at half one’s usual efficiency translates into one workday lost, which is again valued as €278.

Here, it should be noted that there are three measurements at *t*_0_, *t*_1_ and *t*_2_, each 6 months apart. At each time point, the costs of sick leave and work cutback days (*C*_0_, *C*_1_, *C*_2_) were assessed over the last 4 weeks. The area under the curve (AUC) method was used to compute the cumulative costs (CC) over the full follow-up period of 12 months, while interpolating the costs in the months between the measurement points. This was done with the following equation: ${\text{CC}}\, = \, 2C_{0} \, + \, 3\left( {C_{0} \, + \,C_{ 1} } \right)/ 2\, + \, 2C_{ 1} \, + \, 3\left( {C_{ 1} \, + \,C_{ 2} } \right)/ 2\, + \, 2C_{ 2}$.

Finally, the difference between the experimental and control condition in the cumulative costs represents the economic benefits when one condition has lower cumulative costs than the other condition. It was hypothesised that the experimental condition would be associated with lower cumulative costs and hence with monetary benefits.

### Statistical analysis

The analyses were conducted in several steps.

First, we checked for baseline imbalances using descriptive statistics that are presented in Table 1.

**Table 1:** Sample characteristics at baseline

	Waitlist	Intervention
	(*n* = 142)	(*n* = 161)
Female gender, %	98	95
Age, mean (SD)	45 (12.1)	44 (11.1)
Education, %		
Lower	0	1
Intermediate	94	88
Higher	6	11
Years of employment, mean (SD)	3.9 (0.5)	3.9 (0.5)
Job satisfaction, mean (SD)^a^	6.9 (1.5)	6.9 (1.4)
Work hours/week, mean (SD)	25.6 (6.0)	25.6 (6.0)
Work days/week, mean (SD)	3.9 (0.9)	3.9 (1.0)
Costs of absenteeism, € 2014 (SD)^b^	343 (1207)	373 (1282)
Costs of presenteeism, € 2014 (SD)^b^	38 (112)	82 (274)

^a^Job satisfaction: on a scale between 0 and 10; *SD* standard deviation

^b^Costs (in €, 2014) per 4 weeks stemming from productivity losses over time

Second, we adhered to the intention-to-treat principle as per the CONSORT and CHEERS guidelines (Schulz et al. 2010; Husereau et al. 2013). To this end, missing observations at *t*_1_ and *t*_2_ were imputed for which we used regression imputation with predictors of the outcome for precision and with predictors of missingness to account for possible selective dropout. This way, a regression model was estimated to predict the observed values of a variable based on other variables, which was then used to impute values in cases where the value of that variable was missing.

Third, using the imputed costs of absenteeism and presenteeism at *t*_0_, *t*_1_ and *t*_2_, we computed the total annual cumulative costs using the area under the curve methodology as outlined before. Next, we added the intervention costs of €50 per employee for those who were the recipients of the Stress-Prevention@Work intervention.

Fourth, we evaluated the between-group difference in the total cumulative cost (as the outcome of interest) in a regression model with the group variable (0 = control; 1 = experimental) as predictor. The *b*-coefficient belonging to the group variable captures the net benefits, NB, when the cumulative costs of the intervention group are lower than those of the waitlisted control group. The corresponding *t* test belonging to the *b*-coefficient provides a test if the net benefit is statistically significant. It is worth mentioning that the employees are ‘clustered’ in teams and we carried out a design-based regression analysis that took this clustering into account using robust standard errors that were obtained under the first-order Taylor-series linearisation method. In addition, our regression model took into account the non-normality of costs and therefore we employed a non-parametric bootstrapped regression model which was bootstrapped 2500 times.

Finally, with the NBs in hand, we completed the investment appraisal by computing the cost–benefit ratio as C/NB, where *C* is the intervention cost of €50 per employee. We also computed the return-on-investment, ROI, as ROI = NB/C. These metrics, especially NB and ROI, are key to an investment appraisal and serve to decide if investing in the Stress-Prevention@Work implementation strategy is worthwhile as seen from the employer’s business perspective. All analyses were carried out in Stata 14.1 and Microsoft Excel 2010.

### Sensitivity analyses

While our main analysis is based on robust techniques, we also carried out several sensitivity analyses.

First, the study was subject to substantial dropout and this may have affected the cost estimates at *t*_1_ and *t*_2_. In the main analysis, the intention-to-treat analysis was performed using regression imputation (RI) of missing observations at follow-up. In the sensitivity analysis, the intention-to-treat analysis was repeated using linear mixed modelling (LMM), to see if the costs followed the same trajectory over time as estimated under the main analysis.

Second, the main analysis was conducted without adjusting for baseline costs of presenteeism and absenteeism, but there was a small (and statistically insignificant) difference between both conditions. Hence, the main analysis was repeated including costs of presenteeism and absenteeism at baseline as a covariate to adjust for this slight baseline imbalance.

Third, given the relatively high dropout, a sensitivity analysis was conducted in which missing observations were imputed using multiple imputations (5 times) by chained equations with predictive mean matching in which ‘‘real’’ observed values from similar cases are imputed instead of imputing regression estimates. This technique is often used to account for non-normality of data which is often the case for costs.

Last, cumulative costs in the base case were calculated on a relatively conservative basis, where the costs at baseline were assumed to be stable for the first 8 weeks (in favour of waitlisted control condition). Hence, in a sensitivity analysis, cumulative costs were calculated using the following formula: ${\text{CC}}\, = \, 5\left( {C_{0} \, + \,C_{ 1} } \right)/ 2\, + \,C_{ 1} \, + \, 5\left( {C_{ 1} \, + \,C_{ 2} } \right)/ 2\, + \,C_{ 2} .$.

## Results

### Sample at baseline

In total, 473 employees (in 30 teams) were approached. Of these, 221 (in 15 teams) were assigned to the waitlisted control group and another 252 (in 15 teams) to the matched intervention group. At baseline, 143 participants in the control group responded to the baseline questionnaire and 161 in the intervention group. These participants constituted the intention-to-treat sample. However, we had no information about the team membership of one individual in the control condition and that information was required for conducting the design-based regression analysis to account for clustering of employees in teams. Hence, the intention-to-treat sample was brought down to 142 participants. In the intervention group, there were 161 participants. Table 1 presents the sample characteristics at baseline and shows that both cohorts have been matched fairly well, because there were no marked differences between both conditions that would require adjustment in the subsequent economic evaluation, except for the slightly higher costs stemming from presenteeism in the experimental condition. This issue is addressed in the sensitivity analyses below.

### Loss to follow-up

**Fig. 1:**
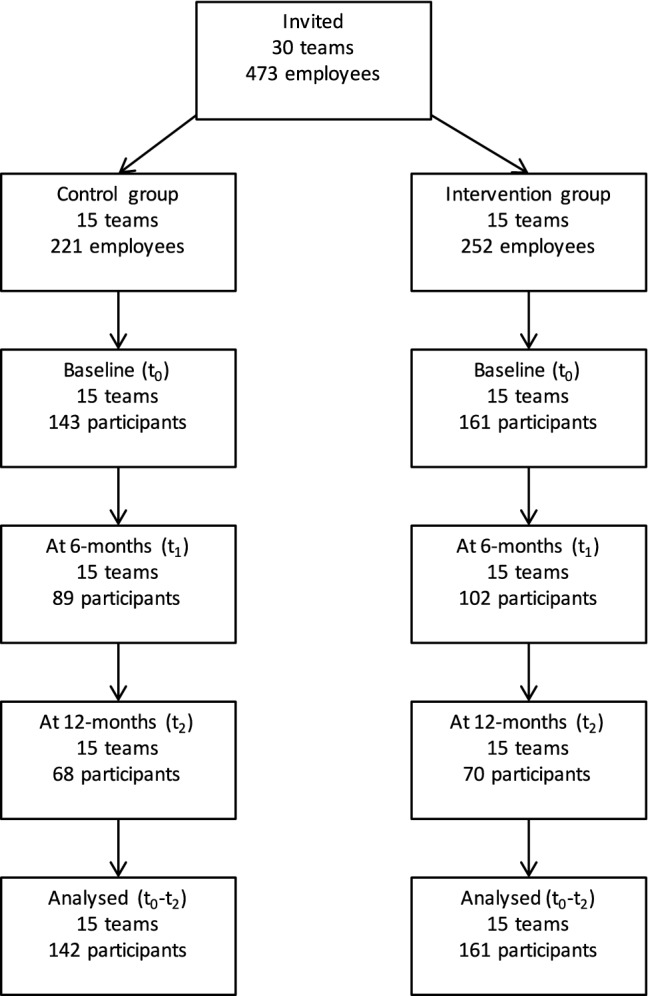
Flow of the participants through the study

The flow of participants through the study is depicted in Fig. 1. Loss to follow-up occurred in both conditions. At the 12-month follow-up 68 (48%) participants were retained in the control group and 70 (43%) in the intervention group. We conducted intention-to-treat analysis and therefore all participants who responded to the baseline questionnaire were included in our analysis except one person in the control group. Missing costs at *t*_1_ and *t*_2_ were imputed using regression imputation as outlined before. Predictors in these regression imputation models were costs of absenteeism and presenteeism at baseline. Other predictors were job satisfaction, female gender and age.

### Costs by condition and time point

Table 2 presents the (imputed) costs (in euro) of productivity losses (stemming from absenteeism and presenteeism in the last 4 weeks) by condition over time. It should be noted that the intervention costs of €50 per employee are not yet included. At baseline, the total costs stemming from productivity losses were somewhat higher in the intervention group (€455) than in the waitlisted group (€381), but in the intervention group these costs decreased much more than was the case in the control group over the 12-month follow-up period. This suggests that there were some cost reductions in the intervention group relative to the control group (especially in absenteeism).

**Table 2:** Costs (in €, 2014) per 4 weeks stemming from productivity losses by condition over time

	Waitlist	Intervention
	*n* = 142	*n* = 161
Baseline		
Absenteeism	343	373
Presenteeism	38	82
Total (95% CI)	381 (212–590)	455 (255–682)
6-month follow-up		
Absenteeism	618	345
Presenteeism	162	175
Total (95% CI)	780 (549–1062)	520 (350–720)
12-month follow-up		
Absenteeism	410	204
Presenteeism	40	88
Total (95% CI)	450 (311–629)	292 (205–423)

*95% CI* 95% confidence interval based on 2500 bootstrap replications

### Cumulative costs over 1 year

Using the total cost estimates of the last 4 weeks at *t*_0_, *t*_1_ and *t*_2_ and employing the AUC method, we computed the annual cumulative costs. Including the intervention costs of €50 per employee in the experimental group, these cumulative costs averaged at €9893 per employee per year in the control group and a lower €6912 in the intervention group.

### Statistical evaluation of the net benefits

The average net benefit is the between-group mean difference of the annual cumulative costs, hence €9893 − €6912 = €2981 (hence in favour of intervention group). The net benefit was statistically evaluated using a bootstrapped and design-based regression model to account for the non-normality of costs and clustering of employees in teams. Under this model the net benefit was estimated to be €2981 (95% CI: − €329 to €6291) per employee per year, which only approached statistical significance (SE = 1689; *z* = 1.77, *p* = 0.078, two-tailed).

### Probabilistic investment appraisal

**Fig. 2:**
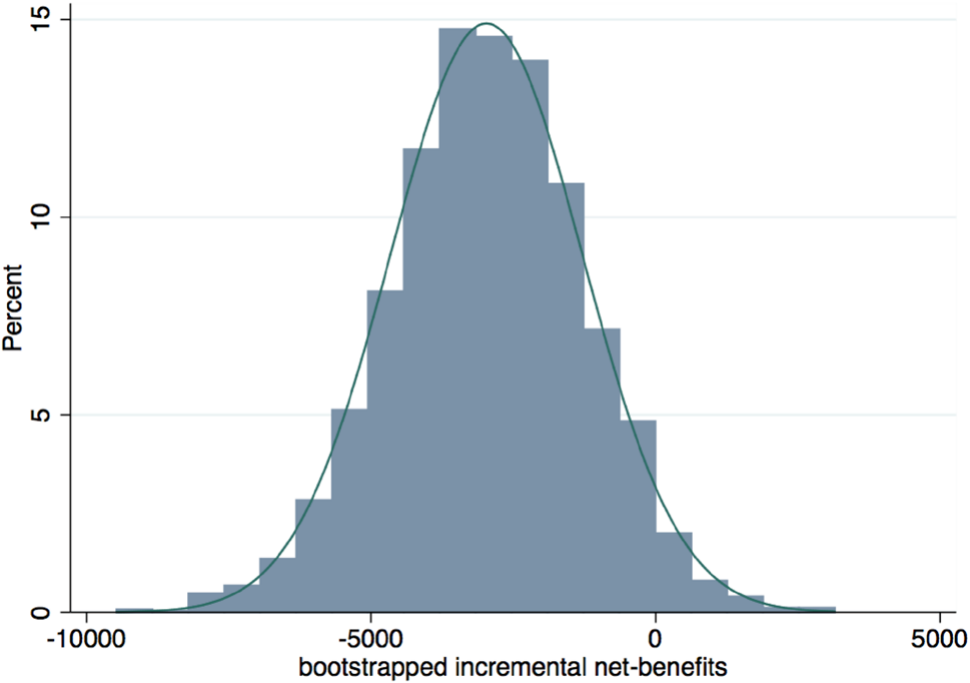
Distribution of bootstrapped net benefits

An investment appraisal aims to support decision-making under uncertainty. The bootstrapped 95% confidence interval of the net benefit was − €329 ~ €6291, suggesting some uncertainty in the estimate of €2981, which is also depicted in Fig. 2.

**Fig. 3:**
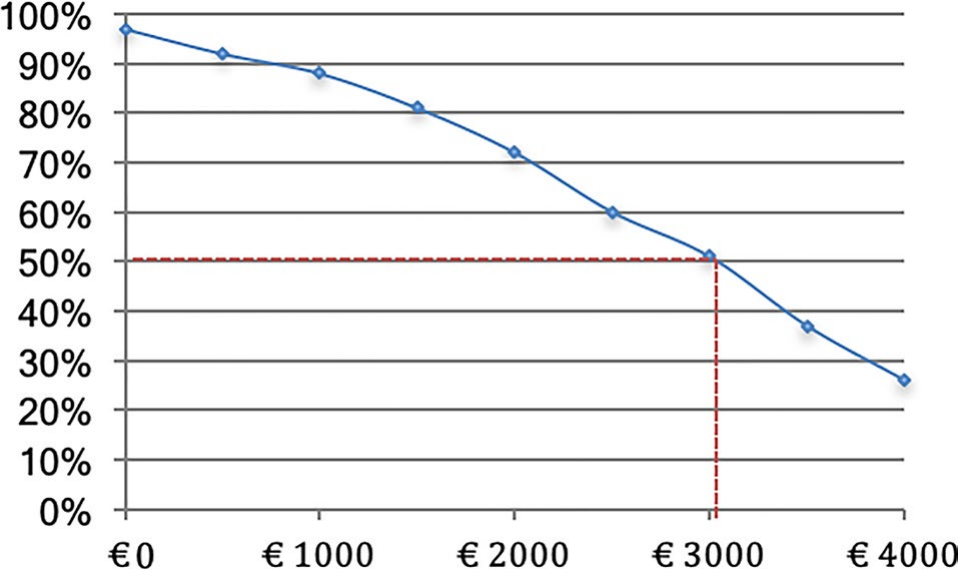
Likelihood (in %) of obtaining at least net benefits of €0 to €4000

However, from a probabilistic perspective, and looking at the distribution of the 2500 bootstrapped net benefits, there is a 96.7% likelihood that the modest investment of €50 will at least break even (be offset) by cost savings within 1 year. Likewise, there is a 92.9% likelihood that the net benefit will be at least €500 (or more), which is a tenfold payout of the initial investment of €50. A net benefit of at least €1000 has still a likelihood of 88.2%. Finally, the mean (approximating the median) expected net benefit of €2981 is associated with a 51% probability. These probabilities (in %) are depicted in Fig. 3 for the various net benefit thresholds (ranging from €0 to €4000), where it can be seen that larger net benefits have a proportionally smaller likelihood of being obtained.

Finally, an NB of €2981 for an initial investment of €50 is equivalent to a cost–benefit ratio of 50/2981 = 0.017 and represents a return-on-investment of 2981/50 = 59.6, i.e., close to 60-fold payout per euro invested.

### Sensitivity analyses

**Fig. 4:**
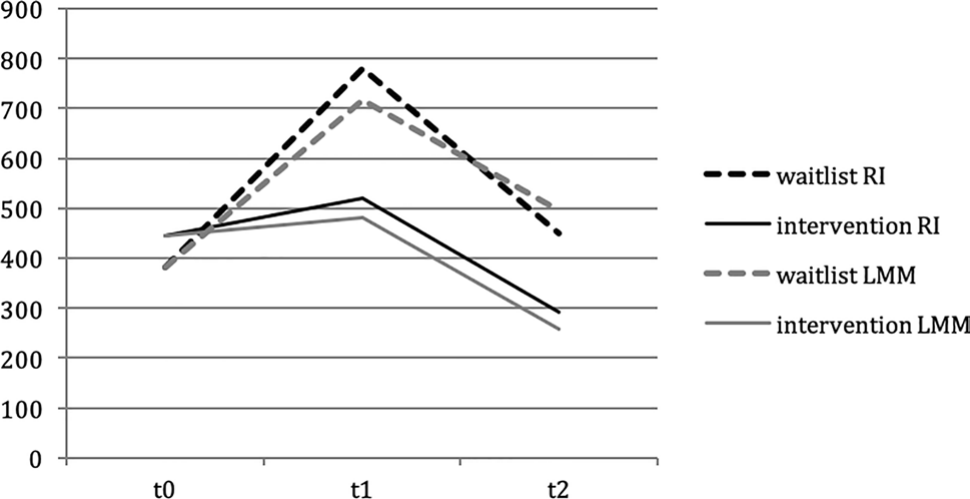
Costs (in €) per last 4 weeks stemming from productivity losses by condition over time—comparing regression imputation (RI) and linear mixed modelling (LMM)

The main analysis relied on regression imputation (RI) of missing observations at follow-up. In the sensitivity analysis, the intention-to-treat analysis was repeated using linear mixed modelling (LMM), to see if the costs followed the same trajectory over time as estimated under the main analysis. The comparison of both analyses (RI and LMM) is depicted in Fig. 4. This shows that the RI and LMM estimates of the costs per condition over the measurement points follow a similar pattern.

As noted before, there was one baseline difference (not statistically significant) that nonetheless attracted attention as a potential confounder: the somewhat higher costs stemming from presenteeism in the experimental condition. In this case, it could be argued that higher baseline costs in the experimental condition would strengthen the null hypothesis that the intervention would not be less costly than the control condition.

Next, the main analysis was repeated, but now with the costs of presenteeism at baseline as a covariate to adjust for possible confounding. In the main analysis the net benefit was estimated at €2981 (95% CI: − €329 to €6291) and after adjustment this increased to €3106 (95% CI: €1344–€4975), which resulted in a statistically significant finding (bootstrapped SE = 938; *z* = 3.31, *p* = 0.001).

Using predictive mean matching (PMM) as imputation technique resulted in a net benefit of €3117 (95% CI: − €530 to €6764), which was not statistically significant (bootstrapped SE = 1861; *z* = 1.68, *p* = 0.094).

The alternative formula to calculate cumulative costs resulted in a net benefit of €3013, which was not statistically significant (bootstrapped SE = 1715; *z* = 1.76, *p* = 0.079).

## Discussion

### Main findings

This economic evaluation was set out to see if the Stress-Prevention@Work implementation strategy among employees of a large health-care organisation represents good value for money as seen from the employer’s perspective. The Stress-Prevention@Work implementation strategy requires an investment of €50 per participating employee. After the intervention, employees in the experimental condition were less often absent from their work and were in addition more productive when compared to matched health-care workers who did not receive the intervention. The greater productivity in the intervention group represented a net benefit of on average €2981 per employee per year when compared to the lesser productivity in the waitlisted control group. This is equivalent to a return-on-investment of close to €60 (rounded) per one euro invested. The outcomes are surrounded by some uncertainty. Nonetheless, there is a likelihood of 96.7% that the initial investment will at least break even after 1 year and there is an 88.2% likelihood that the net benefits will amount to €1000 per employee in a year. Hence, the overall picture is in favour of the Stress-Prevention@Work implementation strategy, which is in line with recent views that (mental) health promotion can benefit workers and employers (Fouquet et al. 2019; Sorensen et al. 2016; Thompson et al. 2018). As Stress-Prevention@Work is mainly a preventive strategy, it is interesting to see positive effects given the relatively short time horizon. This has been the case for other programmes as well (Noben et al. 2014, 2015; Oude Hengel et al. 2014); however, a short time horizon is likely to affect net benefits as worksite health promotion programmes increase per-employee costs at the short term, while the related improvements in productivity solely occur at the longer term (van Dongen et al. 2017). Nonetheless, more than a decade ago, it was concluded that employers and researchers remain largely unaware of the value of quality care and psychiatric intervention, that productivity may increase as a consequence of psychiatric intervention, and that those improvements may offset the cost of the treatment (Goetzel et al. 2002; Langlieb and Kahn 2005).

Not all programmes are equally effective, for example due to poor implementation or low compliance rates (Goetzel et al. 2014; van Dongen et al. 2016). Hence, based on the work of Goetzel et al. (2014), employers considering implementing health promotion programmes should focus on (1) clarifying why the programme needs to be implemented (i.e., do not focus solely on financial gains); (2) ensuring that the programme fits into the culture of the organisation; and (3) ongoing outcome monitoring and evaluation to strengthen implementation (Goetzel et al. 2014).

### Strengths and limitations

This study has some strengths and limitations. Among its strengths, we must mention the use of robust statistical techniques accounting for baseline imbalances, dropout, clustering of employees in their teams and the non-normality of costs. Sensitivity analyses attested to the robustness of the main findings. There are also some limitations that need to be addressed.

First, neither the individual employees nor their teams had been allocated randomly to the control and experimental conditions. Nonetheless, at baseline we did not observe between-group differences that required adjustment in the economic evaluation except costs of presenteeism (and absenteeism) at baseline. A sensitivity analysis adjusting for this possible confounder showed that the net benefits were underestimated in the main analysis and increased from €2981 to €3106, indicating that the main analysis was more conservative.

Second, loss to follow-up was substantial and could have biased the estimates at follow-up. However, we used intention-to-treat analysis by imputing missing observations. This was done with regression imputation with both predictors of outcome (to increase the accuracy of the imputed values) and by predictors of loss to follow-up (to counter the effects of selective dropout). In one sensitivity analysis, linear mixed modelling was used instead and replicated the pattern of how costs stayed much the same in the experimental group between *t*_0_ and *t*_1_ and then dropped at *t*_2_, which contrasts with the control group where costs increased between *t*_0_ and *t*_1_ and then dropped off at *t*_2_. Lastly, an analysis was performed in which missing data were imputed using multiple imputation with PMM. Given that these replications provided similar results, this attested to the robustness of our main analysis. When comparing responders with non-responders (dropouts), non-responders demonstrated higher baseline costs of absenteeism (€389 vs €321), but lower baseline costs of presenteeism (€52 vs €74). Other characteristics (e.g., job satisfaction, days of work per week, hours of work per week, female gender and age) were similar in both groups.

Third, absenteeism and presenteeism were based on self-report over the last month and this may have caused some recall bias. It should be noted that it is hard to see how the costs of presenteeism could have been measured without relying on self-report. Also, it was a conscious choice to keep the recall period short (last 4 weeks) and not, say, the last 3 months to minimise recall bias. Ideally, we would have liked to cross-validate the self-reported data of employees with company-registered sickness absence data. This was, however, not possible due to privacy regulations.

Fourth, stress reduction may also have impacted on staff turnover and the employees’ ability to continue working until the age of retirement. Now, the intervention’s impact on resignation and early retirement remains unknown, as such assessments would have required a much longer follow-up and larger sample size.

Fifth, the effect of the (additional) intervention(s) may be dependent on how these intervention activities are undertaken (i.e., during or after working hours). For example, we have assumed that more extensive interventions take place outside office hours; however, this assumption might be harmful to the level of compliance achieved in implementing the intervention and demotivate participants as the employer invites them to participate in an intervention to reduce work stress and then tells them to do it in their spare time. Hence, it is recommended that employer and employee both keep this in mind and maintain an open conversation. Further studies should examine the extent to which this issue plays an important role in the compliance and effectiveness of more extensive additional interventions.

Sixth, the waitlisted control design may have caused a more beneficial effect of Stress-Prevention@Work compared to the waitlisted control condition, as the use of a waitlisted control condition has been associated with an overestimation of effect sizes in psychotherapy (Furukawa et al. 2014; Cuijpers et al. 2019). Ideally, the control arm would consist of a care as the usual arm, but the waitlisted control was assumed to provide an important incentive to participate in the study. Moreover, Stress-Prevention@Work primarily focused on the organisation instead of individual workers. Although it is possible that organisations in the waitlisted control condition may have postponed implementation of alternative interventions, individuals were not specifically confronted with the results of the allocation process and hence negative feelings associated with the allocation process, which have been argued to be one of the causes of a biased effect size (Furukawa et al. 2014), may have played a minor role.

Lastly, the costs were restricted to the costs of offering the Stress-Prevention@Work implementation strategy, which was the sole purpose of this study. Nonetheless, it should be kept in mind that the Stress-Prevention@Work implementation strategy was designed to encourage the uptake of a stress-reduction intervention—which inevitably introduces costs of its own. These intervention costs have not been included in our analysis, but logic suggests the following: if the per-employee costs of a selected stress intervention would be as high as €2981, then the expected net benefits would cease to exist, and the employer would see a break even between the total costs (of Stress-Prevention@Work plus the stress-reducing intervention) and the benefits (stemming from lesser productivity losses). This suggests that the added per-employee cost of one or another stress intervention can be substantial well before the employer begins to see that the costs begin to exceed the benefits. It should be noted, however, that in the Netherlands, mental health care is most often covered by the health-care insurance (either basic or specialised mental health care).

## Conclusion

There is a business case to be made of encouraging the uptake of a stress-reducing intervention for health-care workers by Stress-Prevention@Work. This implementation strategy costs approximately €50 per employee, but is associated with a high likelihood that these initial investments are more than recouped by the employer because of lesser absenteeism and presenteeism. It should also be kept in mind that the cost of any subsequent stress-reducing intervention has to be subtracted from the expected net benefits of €2981. It is therefore recommended that the Stress-Prevention@Work portal not only offers information that helps to choose from the wide range of available stress-reducing interventions, but in addition should offer information about the per-employee costs of those interventions. That would help to better inform decision-making processes and would in addition help to reduce uncertainty about the health-economic viability and sustainability of stress-prevention at work.

## Data Availability

The datasets used and/or analysed during the current study is available from the corresponding author on reasonable request.
